# Neuropathy Caused by Metal Hypersensitivity after Placement of Stainless Steel Plate

**DOI:** 10.1155/2020/9789021

**Published:** 2020-01-14

**Authors:** Sunyarn Niempoog, Seksan Kukreja

**Affiliations:** Orthopedics Department, Faculty of Medicine, Thammasat University, Thailand

## Abstract

Metal hypersensitivity is a rare complication for implants especially with neuropathy involvement. There was not any previous report suggesting metal hypersensitivity manifested in the form of neuropathy or tendinopathy from metal plate implantation. Here, we present a case of a 42-year-old female with chronic ulnar wrist pain and unremarkable physical and radiological findings. Ulna shortening osteotomy with small stainless steel-made DCP and screw fixation was done. On the third day postoperative, the patient developed pain, swelling, ulnar neuropathy, and flexor tendon contracture. Severe adhesion was found around the implant and the ulnar nerve. Minimal skin patch testing reaction and pathological study suggest a cell-mediated delayed type IV hypersensitivity reaction. A titanium-made LCP was later implanted in place of the stainless steel-made DCP. The patient's clinical status significantly improved after the operation. Metal hypersensitivity in this patient was unprecedented and unique. The severity of the reaction and its location close to the ulnar nerve may predispose to the intensity of the reaction.

## 1. Introduction

Metal hypersensitivity [1–3] is common in orthopedic implants across the body. Hypersensitivity can be either an immediate (within minutes) humeral response or delayed (within hours to days) cell-mediated response. Orthopaedic devices are generally well tolerated, but may sometimes generate corrosion products leading to type-IV delayed hypersensitivity, which is mediated by antigen-presenting cells and T lymphocytes, and can occur either in the postoperative period or months and even years later [4]. Metal hypersensitivity is a rare complication for implants especially with neuropathy involvement. There was a case report of metal hypersensitivity from ulna implant in 1975, implicating cobalt hypersensitivity in cobalt-alloy plates and screws used in the fixation of a fracture of the left radius and ulna. The patient had presented with periprosthetic fibrosis, patchy muscular necrosis, and chronic inflammatory changes seven years after implantation. After removal of all metal implants, the swelling disappeared and the patient's clinical status improved [5, 6]. There was not any previous report suggesting metal hypersensitivity manifested in the form of neuropathy or tendinopathy from metal plate implantation [6]. Impaired wound healing, eczema, sterile osteomyelitis, and tissue swelling were the reported implant-associated allergic reactions from stainless steel plates [7].

## 2. Clinical Course

**Figure 1:**
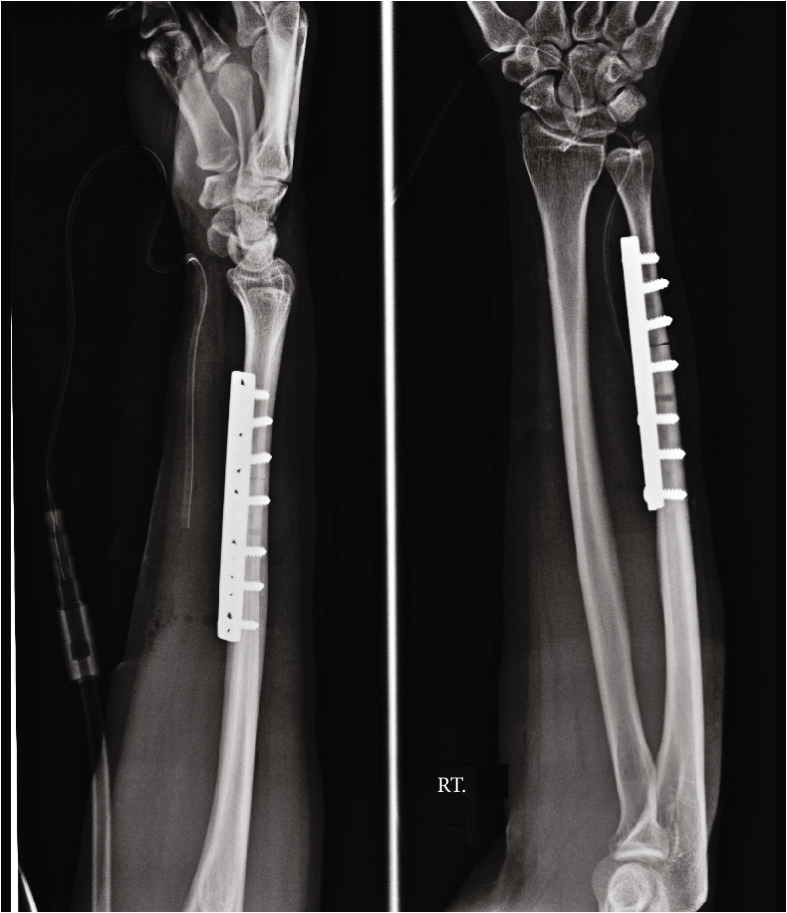
Ulna shortening osteotomy was placed with 3.5 mm small DCP.

**Figure 2:**
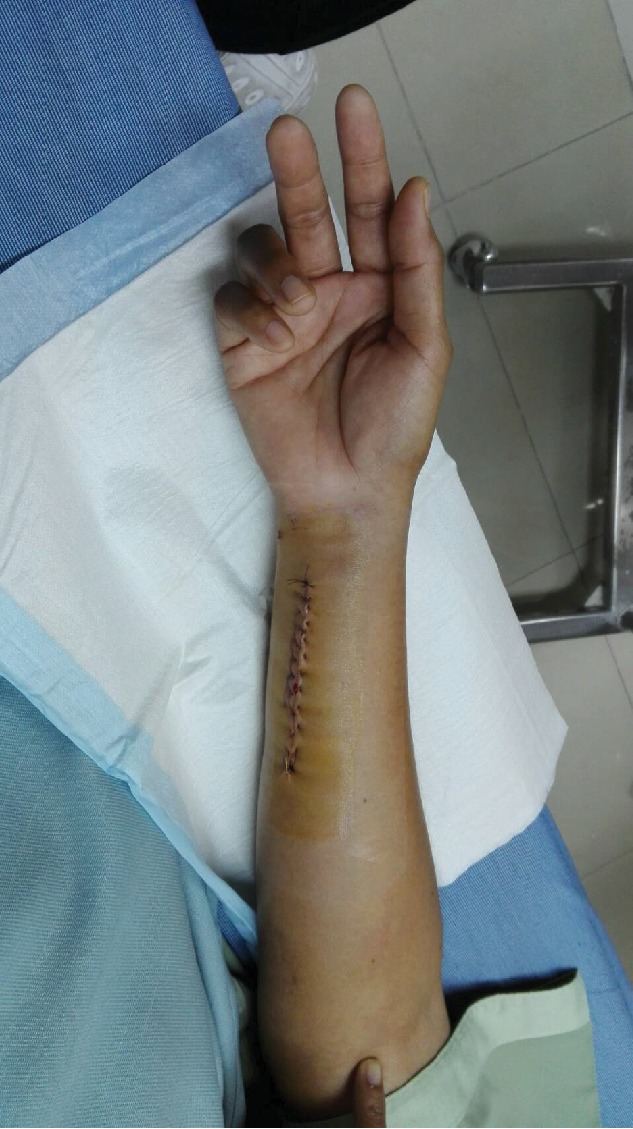
Swelling at the forearm and flexion deformity of the ring and little fingers.

**Figure 3:**
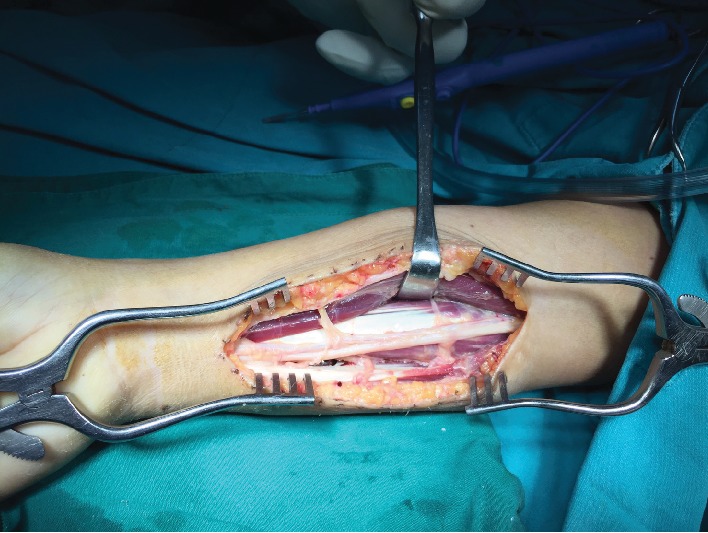
Severe adhesion was found accumulating around the small DCP and the ulnar nerve.

**Figure 4:**
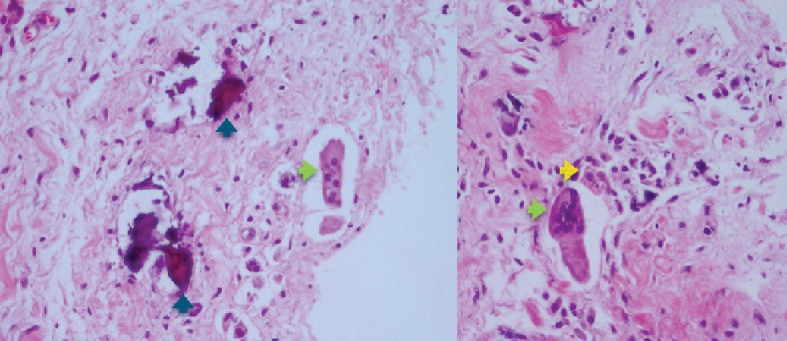
Pathological study from the collected specimen in the second operation. H&E staining; fragments of necrotic bones were identified (blue arrow). There are infiltrations of lymphocytes, histiocytes (yellow arrow), and multinucleated foreign body giant cells (green arrow).

**Figure 5:**
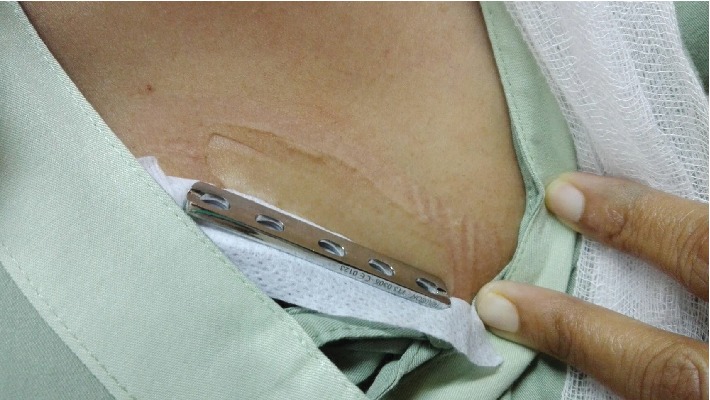
Skin patch testing; the testing material is stainless steel. Minimal local redness, swelling, and eczema were shown.

**Figure 6:**
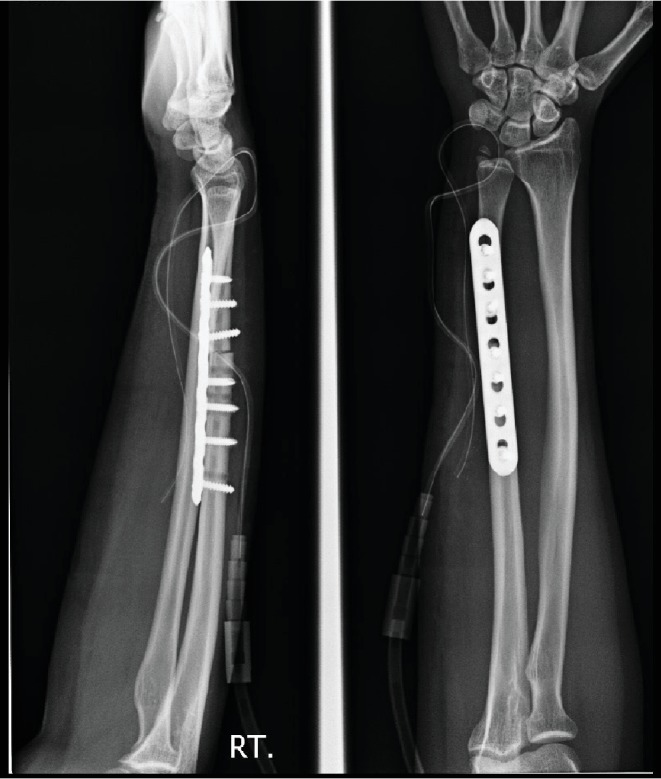
The stainless steel DCP was replaced by 3.5 mm titanium LCP.

**Figure 7:**
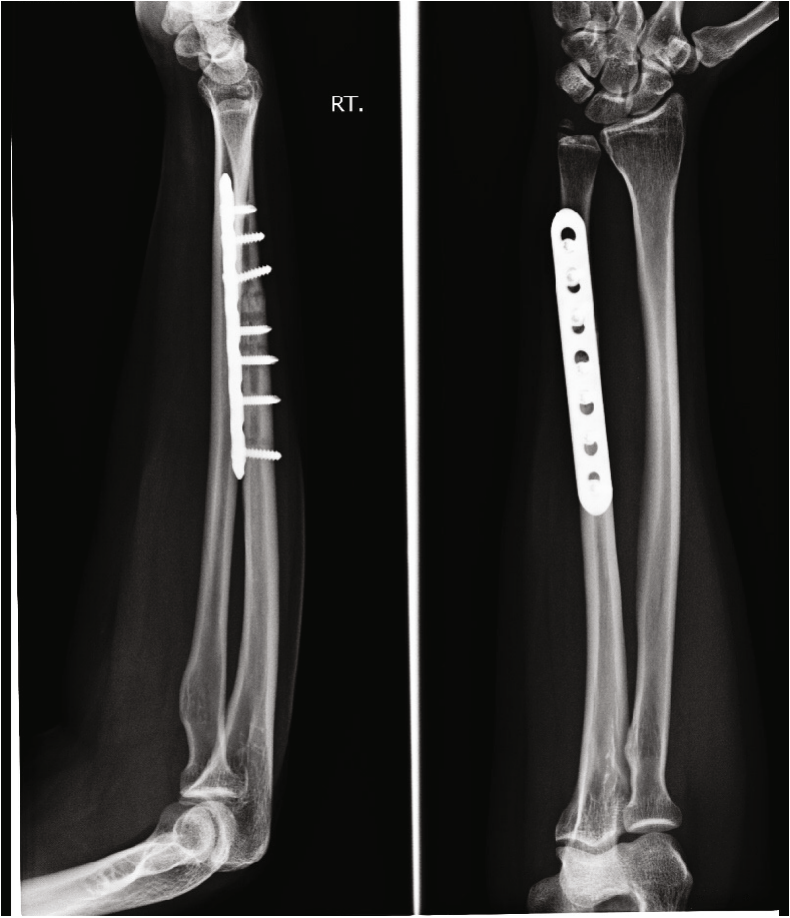
Six months follow-up. Ulna osteotomy was united.

**Figure 8:**
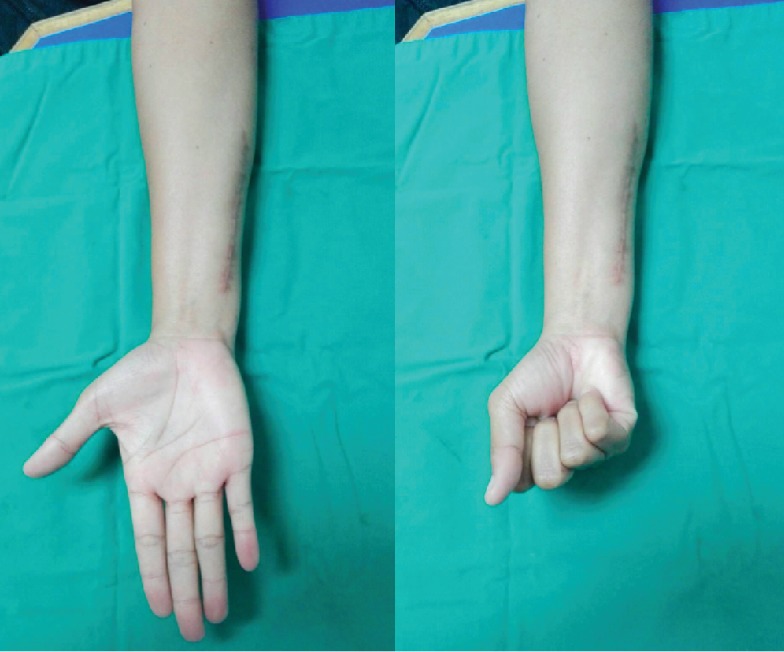
Upon examination, recovery of motor and sensory function of the hand were observed. There were no swelling, tenderness, or neuropathy.

A 42-year-old female patient presented with chronic ulnar wrist pain of the right wrist without preceding injuries and a negative allergy history. Physical examination and initial plain film of the painful site were unremarkable. After failure of conservative treatment with NSAIDs and splinting for more than one year, ulna shortening osteotomy with small stainless steel-made DCP and screw fixation was done (Figures 1 and 2). On the third day postoperative, the patient developed pain and swelling at the surgical site. Upon examination, flexion deformity of the ring and little fingers, limited active range of motion of wrist extension, fourth and fifth finger extension, full passive range of motion, and decreased sensation on the volar surface of the fourth and fifth fingers of the right hand and wrist were observed. Accordingly, the patient was reoperated due to suspicion of ulnar nerve injury. Severe adhesion around the small DCP was found (Figure 3). There was no sign of ulnar nerve, flexor digitorum superficialis, or flexor digitorum profundus injury. The tissue biopsy was collected and sent for further examination, which revealed infiltration of lymphocytes, histiocytes, and multinucleated foreign body giant cells, suggesting a cell-mediated delayed type IV hypersensitivity (Figure 4). Skin patch testing was performed and found minimal reaction (Figure 5). The stainless steel-made DCP was later replaced by a 3.5 mm titanium-made LCP in the third operation (Figure 6). Six months after the third operation, the ulna was union (Figure 7). The patient was symptom-free and able to use the right hand in daily activities (Figure 8).

## 3. Discussion

Metal hypersensitivity is considered in this patient because of signs of inflammation upon day three after the first operation, after which, there was no evidence of infection found from tissue biopsy. Severe adhesion around the metallic device and minimal skin patch testing reaction are the rationale [1, 7]. After replacing the small stainless steel-made DCP with a titanium-made LCP, the patient's clinical status improved significantly. This suggests that the implants are responsible for the reaction. Metal hypersensitivity in this patient was unprecedented and unique. The severity of the reaction and its location close to the ulnar nerve may predispose to the intensity of the reaction. Moreover, introduction of lymphocyte transformation test along with patch testing could also benefit in future operations and in patients susceptible to metal allergies as it would allow for the identification of subjects who are likely to develop implant-related hypersensitivity reactions and to avoid the development of allergies from joint implantation and reveal any reaction due to the implant compounds [4].
